# Supplementary material for: Biochemical dissection of a fungal highly reducing polyketide synthase condensing region reveals basis for acyl group selection
Source: Chem Sci. 2025 Jun 23;16(29):13173–82. doi: 10.1039/d5sc01027f (PMC12199272; doi:10.1039/d5sc01027f)
Supplement: SC-016-D5SC01027F-s001 [file SC-016-D5SC01027F-s001.pdf]

Supplementary Information (SI) for Chemical Science.
This journal is © The Royal Society of Chemistry 2025

# Supporting Information

## Biochemical dissection of a fungal highly reducing polyketide synthase condensing region reveals basis for acyl group selection.

Mia E. Foran[a], Nazia B. Auckloo[a], Y. T. Candace Ho[a], Shaonan Liu[b], Yang Hai[b] and Matthew Jenner[a]*

[a] Department of Chemistry, University of Warwick, Coventry, CV4 7AL, UK

[b] Department of Chemistry and Biochemistry, University of California, Santa Barbara, 93106, USA

* Corresponding author: Matthew Jenner (m.jenner@warwick.ac.uk).

## 1. Supplementary Figures

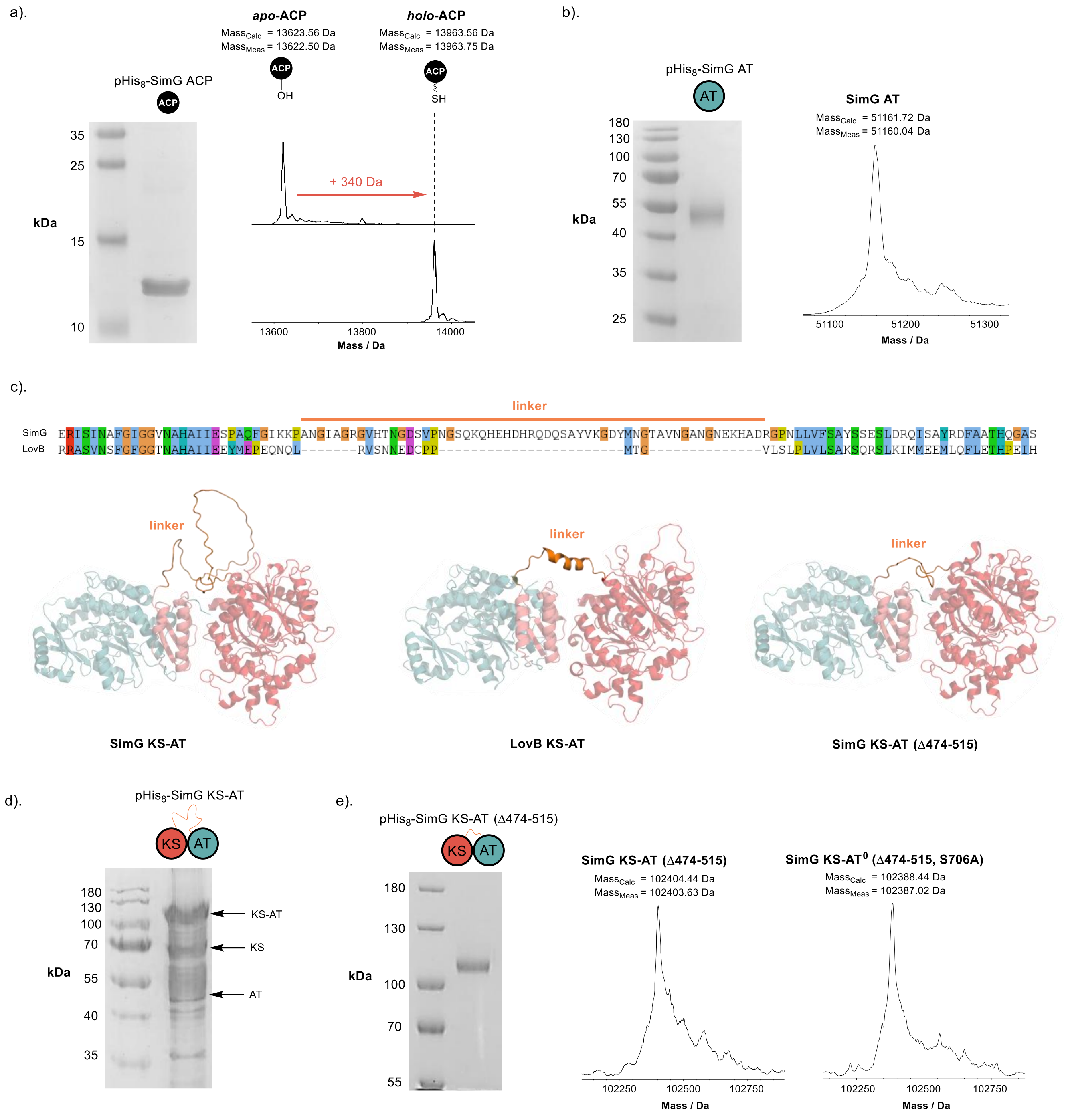


**Supplementary Figure S1.** Biochemical dissection of the SimG hrPKS condensing region. **a**). 12 % SDS-PAGE gel of purified SimG ACP domain (*left*) and deconvoluted mass spectra of *apo*- and *holo*-ACP domain (*right*). **b**). 10 % SDS-PAGE gel of purified SimG AT domain (*left*) and deconvoluted mass spectrum (*right*). **c**). Sequence alignment of the linker region between KS and AT domains in SimG and LovB hrPKSs (*top*) and structural visualisation of the linker regions (*bottom*). The structures of SimG KS-AT and KS-AT (Δ474-515) were generated using AlphaFold 3.[1] **d**). 8 % SDS-PAGE gel of SimG KS-AT didomain showing degradation after $Ni^{2+}$-NTA purification. **e**). 6 % SDS-PAGE gel of purified SimG KS-AT(Δ475-516) (*left*), deconvoluted mass spectra of KS-AT (Δ474-515) (*middle*) and KS-AT (Δ474-515, S706A) (*right*).

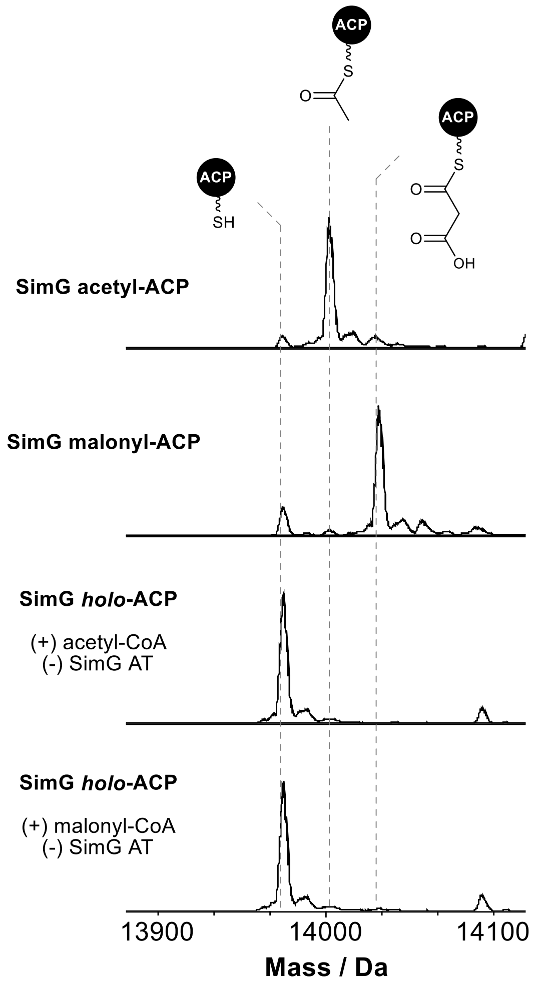


**Supplementary Figure S2.** Deconvoluted mass spectra of SimG ACP species from control reactions of SimG AT-catalysed transfer of acetyl and malonyl units. No transfer of acetyl or malonyl units was observed over the duration of the reactions when the SimG AT domain was omitted.

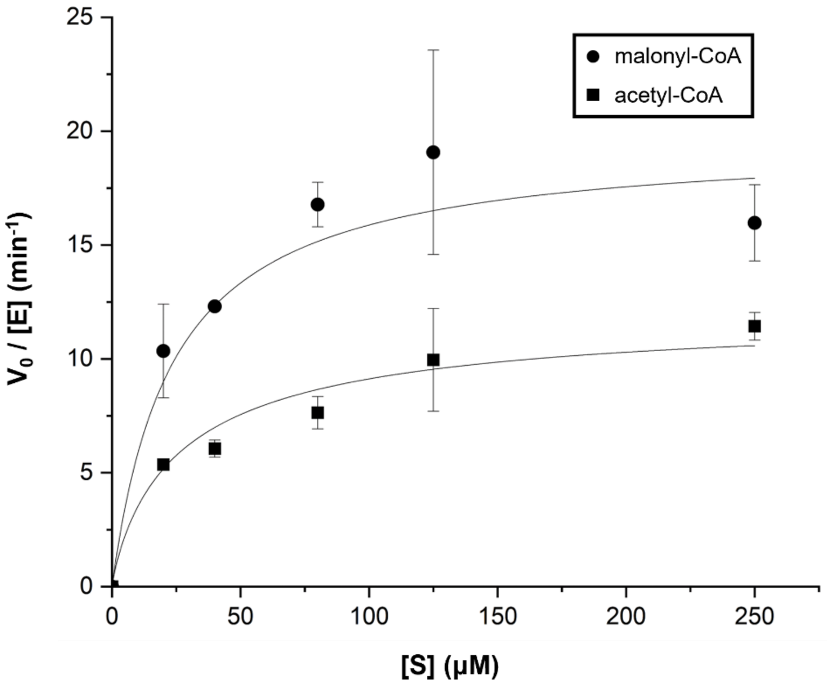


**Supplementary Figure S3.** Michaelis–Menten plot of SimG AT-catalysed transacylation of acetyl- and malonyl-CoA to SimG *holo*-ACP domain. Assay conditions used 1 µM SimG AT domain and 50 µM SimG *holo*-ACP domain, with acyl-CoA concentrations between 20 – 250 µM. The kinetic constants are shown in the main text (**Fig. 3d**).

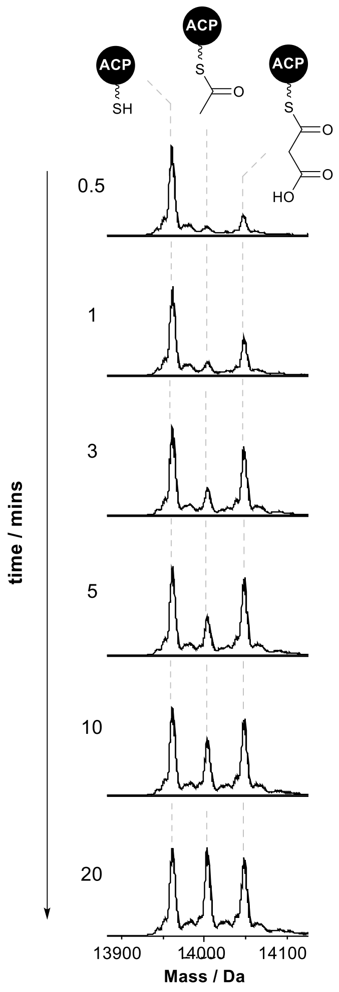


**Supplementary Figure S4.** Deconvoluted mass spectra of SimG ACP species from a time-course analysis of SimG AT-catalysed loading of acetyl- and malonyl- units in a competition reaction. Early time points show preferential loading of malonyl, before accumulation of acetyl units becomes the major species. Faster offloading of malonyl units compared to acetyl units (see **Fig. 4**) allows the acetyl-ACP species to persist over time.

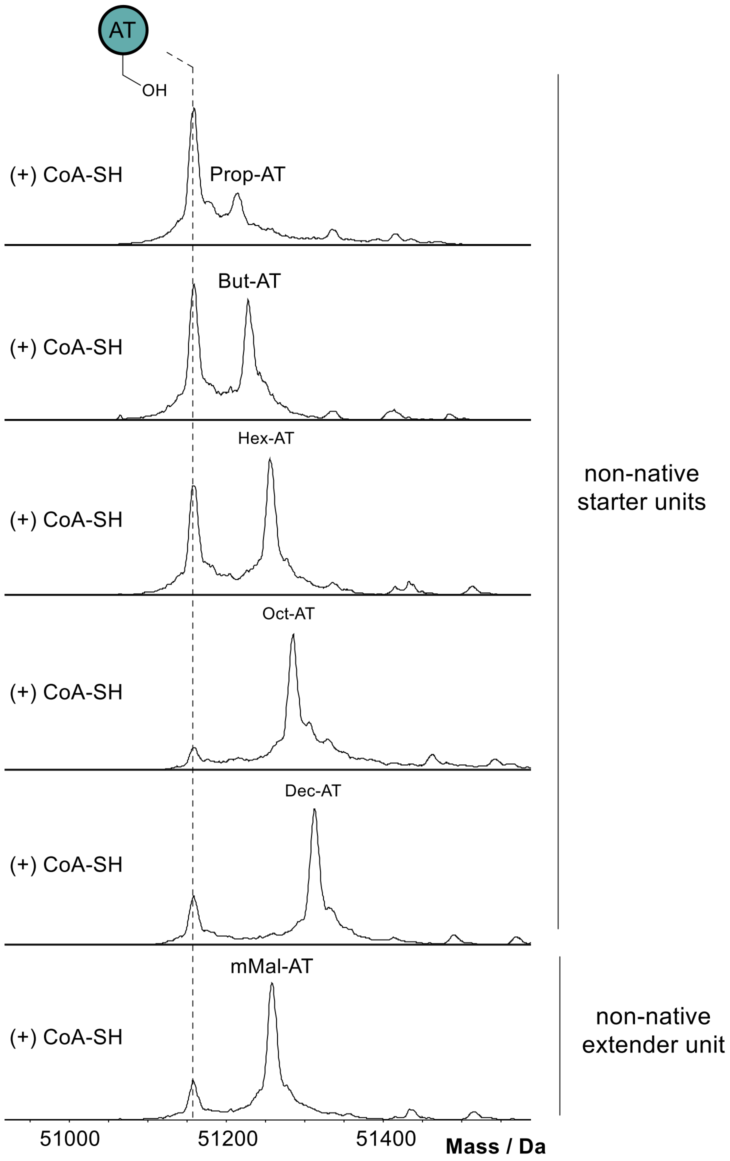


**Supplementary Figure S5.** Deconvoluted mass spectra of non-native acyl-SimG AT species following offloading reactions. A decrease in offloading efficiency is observed as chain length increases for non-native starter units (propionyl-, butyryl-, hexanoyl-, octanoyl-, decanoyl-AT), whilst negligible offloading was observed for the non-native extender unit, methylmalonyl(mMal)-AT.

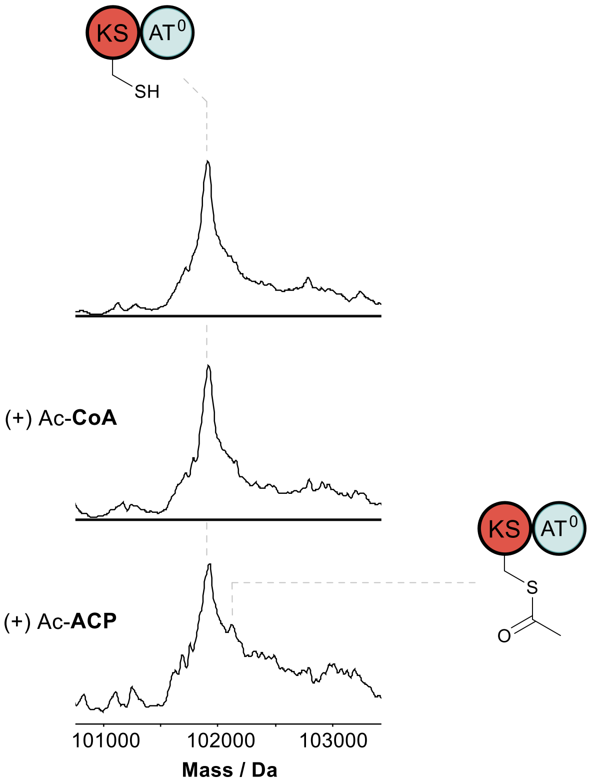


**Supplementary Figure S6.** Deconvoluted mass spectra of SimG KS-AT$^0$ (*top*), following incubation with excess acetyl-CoA (*middle*) and acetyl-ACP (*bottom*). No acetyl transfer to the KS domain is observed using acetyl-CoA, whereas a peak corresponding to +42 Da can be observed when acetyl-ACP is supplied. Full acetylation of the KS domain is likely prevented by competing hydrolysis during the course of the reaction.

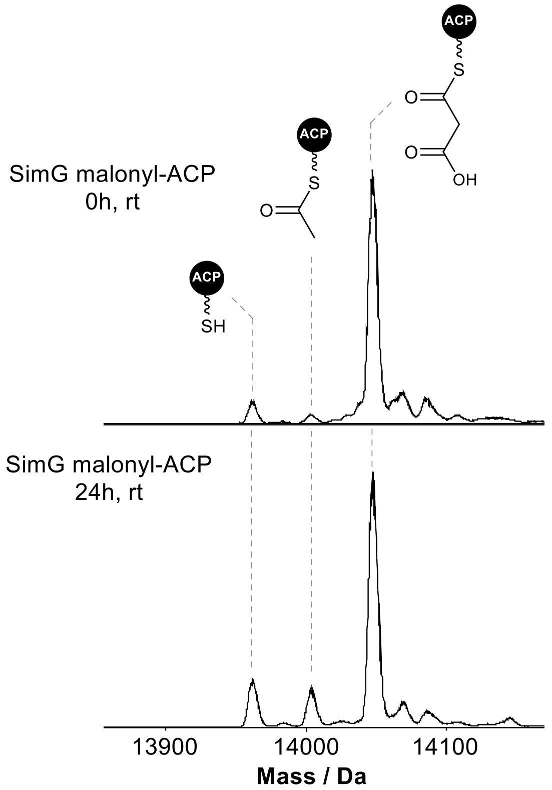


**Supplementary Figure S7.** Deconvoluted mass spectra of malonyl-SimG ACP at 0 h (*top*) and 24 h (*bottom*) incubations in storage buffer (see Methods) at room temperature. Spontaneous decarboxylation of the malonyl-ACP species gives rise to a small amount of acetyl-ACP after 24 h, which can serve as a starter unit.

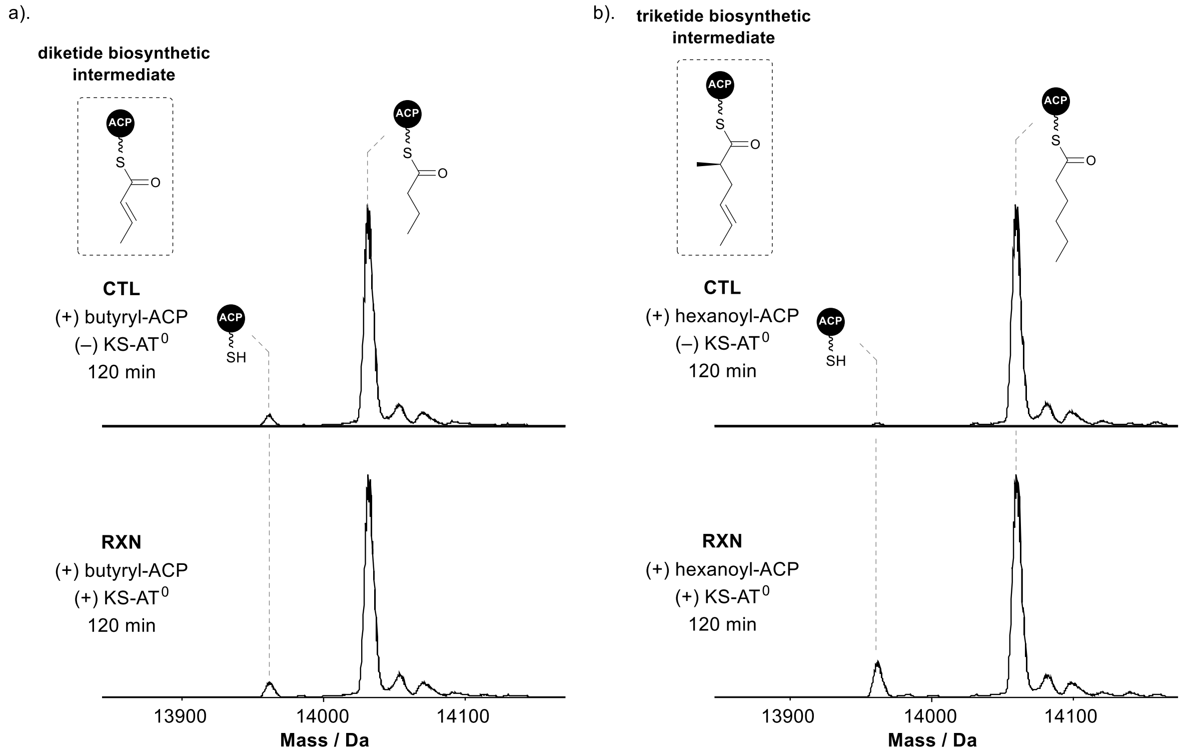


**Supplementary Figure S8.** Deconvoluted mass spectra of **a**). SimG butyryl-ACP domains and **b**). SimG hexanoyl-ACP domain following a 120 min incubation with SimG KS-AT$^0$ (*bottom spectra*) and without SimG KS-AT$^0$ (*top spectra*). Compared to acetyl-ACP (**Fig. 5b**), negligible transfer of butyryl / hexanoyl groups was observed. This may be due to the incorrect functionality of the acyl groups compared to the biosynthetic intermediates (shown in dashed boxes) preventing acylation of the KS domain.

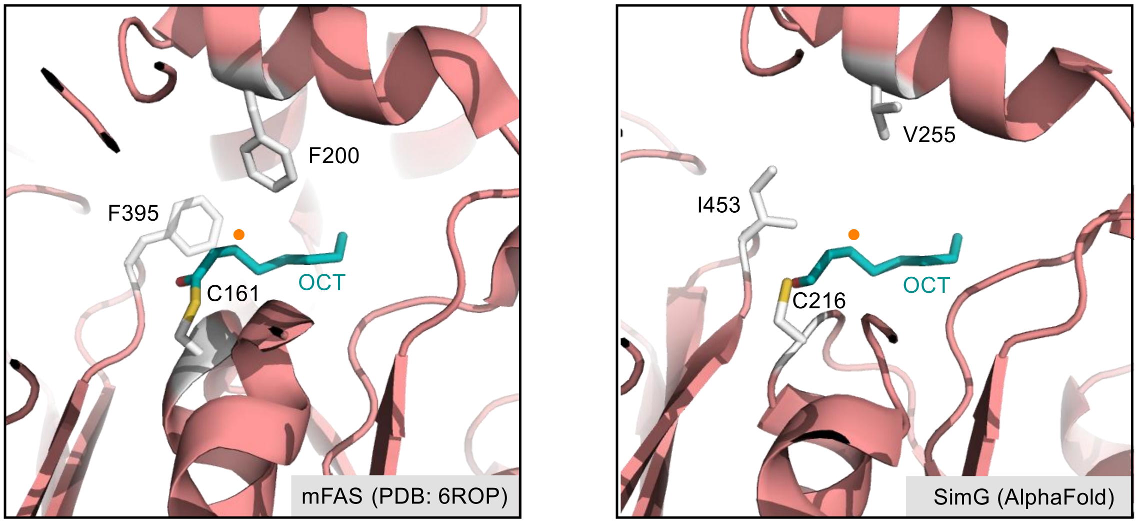


**Supplementary Figure S9.** Structural comparison of KS domains from mFAS (*left*) and SimG (*right*). The mFAS structure (PDB: 6ROP)[2] has an octanoyl group covalently bound to the active site C161 residue, with branching at the β-position (indicated by an orange circle), or a conformationally restricted α,β-unstatured intermediate, prohibited via steric hinderance from F200 and F395. These residues are replaced with V255 and I453 in an AlphaFold 3 model of the SimG KS domain, indicating increased space within the active site.[1]

## 2. Methods

### 2.1. Molecular Cloning and Mutagenesis

#### *2.1.1. SimG hrPKS*

The SimG yeast expression plasmid pXW55-SimG was constructed using *in vivo* yeast recombination cloning. The intron-less SimG gene was cloned from a cDNA library prepared from *Tolypocladium inflatum* NRRL 8044 (GenBank: QEPI01000018.1). Briefly, mRNA was extracted from *T. inflatum* mycelium by using the PureLink™ RNA Mini Kit (Invitrogen) following the manufacturer's instructions. The cDNA library was then syntheszied by using the SuperScript III FirstStrand Synthesis System (Invitrogen) with Oligo-dT primers. The SimG gene fragments were then amplified and assembled with the linearized XW55 vector in *S. cerevisiae* JHY686 strain.[3] The resulting plasmid was recovered by Zymoprep Yeast plasmid miniprep for propagation in *E. coli* XL-10 cells and verified by sequencing.

#### *2.1.2. SimG KS-AT, AT and ACP*

The amplifications of SimG KS-AT didomain, SimG AT domain, and SimG ACP were performed from the full SimG hrPKS construct (2.1.1.) using Q5 DNA polymerase (NEB) and the primers detailed in **Table S2**. PCR products were confirmed on a 1 % agarose gel prior to digestion using NdeI and HindIII restriction enzymes (NEB) and ligation into modified pET28a(+) vector using T4 ligase (NEB) which had also been digested with NdeI and HindIII restriction enzymes (following manufacturers protocols). The modified pET28a(+) vector included two additional His residues in the His-tag and a G2K point mutation to reduce gluconylation of the final protein product (see corresponding protein sequence in **Section 3**). The ligation mixture was used to transform *E. coli* TOP10 cells (Invitrogen) which were plated onto LB agar containing kanamycin (50 µg/mL) and grown at 37 ˚C overnight. Colonies were selected and grown overnight in LB media containing kanamycin (50 µg/mL) prior to plasmid isolation using a miniprep kit (Thermo). The integrity of the gene inserts was confirmed by Sanger Sequencing (Eurofins).

#### *2.1.3. SimG KS-AT (Δ474-515)*

The amplification of SimG KS-AT didomain (Δ474-515) was performed from the SimG KS-AT construct (2.1.2.) using Q5 DNA polymerase and the primers detailed in Table S2. PCR product was confirmed by 1 % agarose gel prior to ligation using a KLD reaction kit (NEB) following manufacturers protocols. The ligation mixture was used to transform *E. coli* DH5α cells (NEB) which were plated onto LB agar containing kanamycin (50 µg/mL) and grown at 37˚C overnight. Colonies were selected and grown overnight in LB media containing kanamycin (50 µg/mL) prior to plasmid isolation using a miniprep kit (Thermo). The desired deletion was confirmed by Sanger Sequencing (Eurofins).

#### *2.1.4. SimG KS-AT$^0$ (Δ474-515), (S706A)*

SimG AT domain containing the S705A point mutation (AT$^0$) was gene synthesised in the pBSK vector (Epoch Life Sciences) for use in this work. The amplification of the SimG AT$^0$ fragment was performed from the gene synthesised construct using Q5 DNA polymerase (NEB) and the primers detailed in **Table S2**. The amplification of the pET28a(+) SimG KS fragment was performed from the SimG KS-AT (Δ474-515) construct (see Section 2.1.3) using Q5 DNA polymerase (NEB) and the primers detailed in **Table S2**. PCR products were separated on a 1 % agarose gel and excised bands were purified using a gel extraction kit (Thermo). The purified PCR products were ligated using a NEBuilder® HiFi DNA Assembly kit (NEB) following manufacturers protocols. The ligation mixture was used to transform *E. coli* DH5α cells (NEB) which were plated onto LB agar containing kanamycin (50 µg/mL) and grown at 37˚C overnight. Colonies were selected and grown overnight in LB media containing kanamycin (50 µg/mL) prior to plasmid isolation using a miniprep kit (Thermo). The presence of the point mutation was confirmed by Sanger Sequencing (Eurofins).

### 2.2. Production of 3*R*-hydroxyl-4*R*-methyl-6*E*-octenoic acid in *S. cerevisiae* JHY686

For the production of 3*R*-hydroxyl-4*R*-methyl-6*E*-octenoic acid, *S. cerevisiae* JHY686 strain[3] harboring the pXW55-SimG plasmid was inoculated to 4 mL of Yeast Synthetic Drop-Out medium without uracil. The cells were grown for 72 hours with constant shaking at 28 ºC. A 1 mL aliquot of the seed culture was inoculated to 1 L YPD medium supplemented with 1% dextrose. The cultures were shaken at 28 ºC for 72 hours. The cells were removed by centrifugation and the liquid medium was acidified with formic acid, followed by extraction with ethyl acetate. LC-ESI-MS analysis of the extract was performed on a Shimadzu 2020 LC–MS system (Shimadzu Scientific Instruments) using a Phenomenex Kinetex 1.7 µm, 2.0 x 100 mm, $C_{18}$ column in positive and negative mode electrospray ionization. The organic layer was concentrated *in vacuo* and the title compound was purified by Biotage Isolera chromatographic system.

### 2.3. Protein overexpression and purification

An aliquot of chemically competent *E. coli* BL21 Star (DE3) cells (50 µL) was transformed with relevant plasmid DNA. Transformed cells were plated onto LB agar plates containing kanamycin (50 µg/mL) and incubated overnight at 37˚C. A single colony was picked and used to inoculate LB media (10 mL) containing kanamycin (50 µg/mL) and incubated overnight at 37˚C with shaking (180 rpm). The overnight culture was used to inoculate a flask of LB media (1 L) with kanamycin (50 µg/mL) which was left to grow at 37˚C with shaking (180 rpm) until an $OD_{600}$ of 0.7 - 1.0 was reached. Protein expression was induced by the addition of IPTG (250 µM) and incubated at 15˚C with shaking (180 rpm) overnight.

Cells were centrifuged (4000 rpm, 15 minutes, 4 ˚C) and resuspended in loading buffer (20 mM Tris HCl, 100 mM NaCl, 20 mM imidazole, pH 8.0). The cells were lysed by high pressure (20 psi) cell disruption and centrifuged (17,000 rpm, 30 minutes, 4˚C) to pellet the insoluble cell components. The cell lysate was filtered through a 0.45 µm syringe filter and loaded onto a 1 mL HiTrap™ Nickel FastFlow column. Following a wash with loading buffer (10 mL), the protein was eluted from the column with buffers of increasing imidazole concentrations (5 mL of 50 mM, 3 mL of 100 mM, 3 mL of 200 mM, 3 mL of 300 mM). Fractions were checked for the protein of interest by SDS-PAGE and those containing protein were concentrated and exchanged into storage buffer (20 mM Tris HCl, 100 mM NaCl, pH 7.6) using an appropriate size Vivaspin centrifugal concentrator (4000 rpm, 4 ˚C). Once concentrated to approx. 1 mL, 50 µL aliquots of protein were flash frozen in liquid $N_2$ and stored at -80 ˚C.

### 2.4. Conversion of *apo*-SimG ACP to *holo*- / acyl-SimG ACP

SimG ACP (200 µM) was converted to its *holo*- or acyl- form by incubation in storage buffer (20 mM Tris base, 100 mM NaCl, pH 7.6) with 10 mM $MgCl_2$, 2 µM Sfp PPTase, and 1 mM CoA / acyl-CoA in a total of 50 µL for 30 minutes at 25 ˚C. For use in **Sections 2.7 – 2.10**, the generated ACP species as diluted 5-fold with storage buffer and concentrated to approx. 50 µL using a 10 kDa MWCO Vivaspin centrifugal concentrator (12000 rpm, 4 ˚C) to reduce the amount of CoA present in the subsequent assay.

### 2.5. SimG AT acylation and ACP transfer

To investigate AT acylation, SimG AT domain (50 µM) was incubated in storage buffer (20 mM Tris base, 100 mM NaCl, pH 7.6) with 1 mM acyl-CoA in 50 µL at 25 ˚C. The reaction was quenched with 1 % formic acid at 1 minute following AT domain incubation. Samples were diluted 5-fold with milliQ $H_2O$ and analysed by UHPLC-ESI-Q-TOF-MS. To capture transfer to the ACP domain, SimG *holo*-ACP (50 µM) was incubated in storage buffer (20 mM Tris HCl, 100 mM NaCl, pH 7.6) with 1 mM acyl-CoA and 10 µM AT domain in a total of 50 µL at 25 ˚C. The reaction was quenched with 1 % formic acid at 1 minute following AT domain incubation. Samples were diluted 5-fold with milliQ $H_2O$ and analysed by UHPLC-ESI-Q-TOF-MS.

### 2.6. Kinetic analysis of SimG AT-catalysed ACP transacylation

SimG *holo*-ACP domain (50 µM) was incubated with SimG AT domain (1 µM) and acyl-CoA (20 - 250 µM) in storage buffer (20 mM Tris base, 100 mM NaCl, pH 7.6), and made up to a final volume of 50 µL at 25 ºC. The reaction was quenched with formic acid (1 % v/v final) at 0.5, 1, 2, 3, 5, and 10 minutes following AT domain incubation before preparation for analysis by UHPLC-ESI-Q-TOF-MS. Raw mass spectra were subjected to

deconvolution (Bruker MaxEnt), taking an average of all charge states. Using the raw peak intensities for *holo*-ACP and acyl-ACP, percentage conversion to the acyl-ACP domain species was calculated using the following equation:

$$\%\ acyl\text{-}ACP = \frac{Inten._{acyl\text{-}ACP}}{(Inten._{holo\text{-}ACP} + Inten._{acyl\text{-}ACP})}$$

Percentage acylation values were converted to concentration, and analysis at each acyl-CoA concentration was completed in triplicate. The gradient of the linear region was determined for each transacylation reaction, which was used to determine the initial rate of transacylation, $V_0$. This initial rate data was set relative to AT domain concentration (1 µM), plotted against acyl-CoA concentration, and fitted to a Michaelis-Menten function (Hill function) in OriginPro, which determined values for the kinetic constants of limiting rate ($V_{max}$), catalytic rate constant ($k_{cat}$), and the Michaelis constant ($K_m$).

### 2.7. SimG AT substrate offloading

SimG acetyl-ACP or malonyl-ACP (50 µM) was incubated in storage buffer (20 mM Tris base, 100 mM NaCl, pH 7.6) with 1 mM CoA and 10 µM AT domain in a total of 50 µL at 25 ˚C. Reactions were quenched with 1 % formic acid after 1 minute following AT domain incubation. Samples were diluted 5-fold with milliQ $H_2O$ and analysed by UHPLC-ESI-Q-TOF-MS.

### 2.8. SimG AT acyl-CoA competition

SimG *holo*-ACP (50 µM) was incubated in storage buffer (20 mM Tris HCl, 100 mM NaCl, pH 7.6) with 125 µM acetyl-CoA, 125 µM malonyl-CoA and 1 µM AT domain in a total of 100 µL at 25 ˚C. The reaction was quenched by addition of 1 % formic acid at 0.5, 1, 3, 5, 10, and 20 minutes following AT domain incubation. Samples were diluted 5-fold with milliQ $H_2O$ and analysed by UHPLC-ESI-Q-TOF-MS.

### 2.9. SimG KS-AT$^0$ acyl-ACP chain transfer

SimG acetyl / malonyl / butyryl / hexanoyl-ACP (50 µM) was incubated in storage buffer (20 mM Tris HCl, 100 mM NaCl, pH 7.6) with KS-AT$^0$ (50 µM) in a total of 100 µL at 25 ˚C. For acetyl-ACP and malonyl-ACP, the reaction was quenched with 1 % formic acid at 0, 30, 60, 90, and 120 mins following KS-AT$^0$ incubation, and butyryl- / hexanoyl-ACP reactions after 120 mins. Samples were diluted 5-fold with milliQ $H_2O$ and analysed by UHPLC-ESI-Q-TOF-MS.

### 2.10. SimG acyl-ACP chain extension

SimG malonyl-ACP (50 µM) and acetyl-ACP (50 µM) were incubated in storage buffer (20 mM Tris HCl, 100 mM NaCl, pH 7.6) with KS-AT$^0$ (100 µM) in a total of 50 µL for 30 minutes at 25 ˚C. Samples were diluted 10-fold in milliQ $H_2O$ and analysed by UHPLC-ESI-Q-TOF-MS.

# 3. Sequences, Tables and Accession Numbers

## 3.1 DNA Sequences

**>simG_hrPKS (START codon removed when cloned into XW55 vector)**

```
ATGGCTCCTCAACAAATATCACCCCCCAATGGGACGGCATCCCCAGAGGCCGGGACTCCAAGATATACGTACAGACCAGCTTCACCTGCACCAGAATCGCCATGGGCAGACAGCGCGG
AAGGGGCCGAGTTGGCCAAGCCCATGGCCATCATCGGCATGGCCATGCGTCTCCCGGGAAGCGTACGCTCGGGTGACGACCTCTGGGACCTCCTCTCCACGAGGAAGAGCGGACTCTG
CGATATCCCCAAGAACCGGTTCAACGCCGACGGGTTCTACGACCCGGCTCGGGGCCCCGGGACGATCCCGGTCAAGAAGGGATATTTCCTCCATGACGTGCAGATCGAGGAGTTCGAC
ACCAACGTCTTCCCCATCCCCAAGATGGAGCTGGAGCGCCTGGATCCTGCCCAGCGACAGTTGCTCCAGGTAGCCTACGAGTGCATAGAGAACGCAGGCGGGACCTCGTGGCGTGGGA
GCAGAACTGGGTGCTACATCGGCGAGTTCGGCGAGGACTTTGCCGACAGTTCTGCACGCGAGTCACAGCAAAGGGGCAACTTACGATACACCGGCCTTGCCGATTTCGCAATAGCCAA
CAGGATCTCGTATGAGCTCGACCTTCAAGGCCCGAGTATGGTGGTCAAGACGGCTTGCTCGTCTTCCCTCGTGTGCCTCGACCTGGCCTGCAAGGCCATCCAGAGCGGCGAATGCGAA
AGTGCCCTCGTCGGCGGCGTCAGTATGCTCTTCTCGCCCGCCACGTACATCAGCGTGACCGACCTGGGCGTCATCTCGCCCAACGGCCAGTGTCGGAGCTTCGACGCCGGCGCGGACG
GCTACGCGCGAGGGGAGGCCGTCAACATGGTCATGATCAAGAGGCTAGATCACGCTCTGAGGGACAAGGACCCCATCCGTGCCGTCATCCGCGGGACCGGTGTCAACACCGACGGCAG
AACCAACGGCATGCTGACCCCAAGCTCCGCCGTGCAAGCGGATCTCATCCGGCACACATACGAGATCGCTGGAATAGATGACCTGTCACAAACCGCCGTCGTGGAGTGTCACGGCACC
GGAACTCCGGTCGGCGACCCCATCGAGACGGAGGCTGTCGGACGGTGCTTTGGCGACGACGGCGTTACCATCACATCAGTTAAACCAAATCTTGGACACGCTGAAGCTGCCGCCGGCC
TGTCCAGCCTGATCAAGTGTGTCCTGAGCTTGGAGCACCGGCAAGTGCTGCCAAATATCAACTTCGAGACACCGAATCCAGACATTCCGTTTGAGAAATACAAACTCCGCGTCCCGAC
CGAGGTTGAATCTTGGCCGCAGGGTCGAGCAGAGAGGATAAGCATCAACGCCTTCGGCATCGGAGGCGTCAACGCGCACGCAATCATCGAATCCCCGGCGCAGTTTGGAATCAAGAAG
CCTGCCAACGGCATCGCCGGCCGCGGCGTACACACGAACGGCGATTCTGTTCCCAATGGTTCTCAAAAGCAGCATGAGCATGATCACCGTCAAGACCAAAGCGCCTACGTGAAGGGCG
ATTATATGAACGGCACTGCCGTCAACGGGGCAAACGGCAACGAAAAGCATGCTGACCGCGGACCGAATCTCCTGGTCTTCTCCGCCTATAGCTCCGAGTCTCTCGATCGGCAGATCAG
TGCTTACCGTGACTTTGCAGCAACTCACCAAGGCGCCTCGCTCAAGGATCTGGCATATACCTTGTCGAGTCGGCGCGATCACAGGCCCTACCGGGCTTATGCCATCGCCGACGACGCA
TCCGGTGTCCAAGACGCCTCCGCCACCGTTAACGCCGTCGTAGACGGAGCACCTCCGCCGGTGGGCTGGATCTTCACCGGACAGGGTGCGCAGTGGCCGGAGATGGGCGCCAGGCTGA
TCGATACCAGTGCTGCGTTCAGAAACAGGATCCGTAAGCTGGACAAGTACCTGCAGACGCTGAAGCTGGAGCCACCGGTGAGCATTGAAGCCGAGCTGCGCAAGACCAAGGAAGACAG
TCGAGTGCACCGTCCCGAGATGGGACATCTCGTATGCGTCGCCATGCAGGTAGCCCTGGTCGACGTCCTCCGCTCCTGGAACATCGTCCCAGACTTTGTCCTGGGCCACTCGTCGGGC
GAAACAGCCGCCGCCTACGCCTGCGGAGCCATCACGGCGGAGGCTGCCGTATACGCGGCAACGAGACGAGGCATCGGCAACGCATCTTCCCAGAGGAAAGGCTCCATGGCTGCCGTCG
GGCTGGGCAGGGATGAGATACAGCCGTTCCTGCTCGAGGGGGTCAACATCGCCTGCGAGAACAGTCAGAGCAACGTCACGCTGTCGGGAGATACGGAGCAGGTTGAGAGCATCGTAGC
GACGCTCAAGGCCGAGAGACCGGGGGTCTTTGCTCGTTTGCTCCGGGTAGAGAAGGCGTATCACTCCCATCACATGTGTGAATACGGGCCGGTGTATGAGGAGCAGCTGAAGCCCGTG
GTTCGCAGCGCTGACCCCGAGATCCCATTCTACTCCTCCGTCACCGGTGACCGCCTGACCGGCGAGGGACAGCTCGGGGCATCGTATTGGCGGGCGAACATGGAGAACACGGTTCTCT
TCAACCCGGCGCTCCGCTCCGCCCTCCGAGATCGACCCGGAAAGCTGGTCCTAATCGAGTTGGGACCTCATCCCGCCCTTGAAGGACCAGTCGGCCAGATTCTCCGAGACCTGGGTCG
CACAGACGACATCTACCTTGGGACCTGTATCAGGGGCAGCGAGTGCGATCGCAGCCTGCTTCACCTCGGCGGAAAGCTGTACCAGCAGTCGATACCGATGGACCTTGCAGCGATCTGC
CCGCCCGGCGCCGTCTTGACGAACCTGCCGCGCTATGCCTGGAGCCAGGACACGACGCACTGGGAAGAATCCAGGGTTTCGCGCGGCTGGAGGCTCCGGGAACATCCACCCCACGAGC
TTCTCGGGAGCAGAGTGCTGGAGACGGAGGGCGAGCCGTGTTGGAGAAAGGTAATGGCCCTCGAGGACGCTCTCTGGCTGGATGGCCACGAGGTCAACGGCCAGGTCCTGTTCCCGGC
TGCTGGATACATCTCCATGGTAGGCGAGGCGCTCCGACAGCTGACCGGCGACGCGACATTCACCGTCAGGAACGTCCGCATCATGTCGGGCCTGGTGCTGAGCTCGGACAAGCCGGTC
GAGCTGGTCACTCTATTGAGGTCGACCACGATGGGCACCTCGGACGATTCGGAATGGTACGAGTTCACCATCACCTCGTTCGACGGCACGGCATGGGTCCGGAACTGCCACGGCGAGG
CCATGGCGTCAAGCGACAAGTCGTTTCACCTCGACTCTGTCTCTCCTGCAGCAGGATCTTTCCCTCGCAAGCTTGACCGGAGGGACTCTTACGACATCCTGAGACGAGTTGGCTTCAA
CTACACCGGGGAGTTTGAAGGGATGGCGGACATCTCGGTCTCGCCGTCGTCGCTTCAAGCCCACGGCGTCACTTCCCCGGCTCGCCAAGTGACCGACAGGCCGTCCAGGTGCGCTACG
TACTCGGTGCACCCGGCGGTGATCGATCAATGCTTCCAGCTCTTCACGGTGGCCCTATGCCGCGGACTCAGGCGCAGCAACAGGCGGCTGGCGGTCCCGACCTTCATCGAGGAGCTGG
TCGTGTCGCCGAGCGCATCGCCCCTGAGCGTCACCGCCAAGATCAACCGCCTGGACGAGGTGGGATCCTGGACAGGCGATTTCGTTGCCCTCGCGGCCGAGGGGCCCGTCGTGAGGCT
CAAGGGGATGAAGTCCGTCGTCCTCGCGACCCCGGCGCAGTCAGAGCCGCCCCTATCCACGGAACTCGAGTGGAAGCCGCACAGCGACTTTGTCGGATTGGGGGTCGGCCTGCACCCG
CGTGTGCCAAGGAAAAGGGAGTGGGCTCTGCTGGAGGAGCTCGTCCTGCTGTGCAATCTAGACCATCTCGGCCAGATCAAGACCGATGACAGCACTGCGCCGTATCTCATCAAGCTCC
TGGACTGGATGCGCCTTGTTACGGATCGATACAGGTCCGGCAAGAACCTGTTCGTGTCTGCAGGCGCTGGCCTCGAACATCTGGATCACGACGAGCGAGTCGCCAGGATCGATTTCCT
CATGGCCCAACTTGCTGACACGCAGGACGTTGTCTTCGCCAGGGCGATCCATCGCCTCTTCATCGAGGCGCCCTCCATATTCGCGGGAGAGGGCCATCCCTTGCACATCCTCATGCCC
GACAATGTTCTGAGCGACTTTTACGACGCCACATCCTTCGACATGGCCGATGCCGTCCGCCTGGTGGCCAACACGAACCCTCACATGCGCGTGCTCGAGGTTGGCGCGGGAACTGGCA
GCATGACGGCCAGGCTTCTCCGCGCCTTGACGTCCTCCCACGGCGAGCGCCTCTACAGCCAGTACGGGTACACTGATGTGTCGGCCGGCTTCATGGCCGTGGGGAAGCAGCGCTTTGC
CGCCGTCGAGAACATCGATTACGATGTGCTGGACATATCGAAGGATCCCAAGGAGCAGGGATTCAGGCCCGGCAGCTATGATCTCGTTATCGCTTCCAACGTTCTGCACGCTACCCCT
ACTCTGGATGAGACACTTCGCAACGTCTATAGTCTATTGAAGCCCAGTGGACGCTTGTTCCTGGAGGAACTCACTCCAGATGCAATGTTCGTAAACTACGTCATGGGCTTCTTCTACG
GCTGGTGGCTCGGTGCCGAGGACAATCGCGTGGAGCAGCCATGGATCGCGCCCGAATGCTGGGCTGAGAAGTTGGTCGCCGCGGGATTCCAGAAGCCCGAAGCCATCGTTCTGGACAG
CGACAAGCCATATCAGATCAGCGCGGGGATCATCGCTTCACGGGGGGTACGATCGACCTTGCCCTCCAAGGTGGCGGTTCTGTGCCATACCGCCGATGAGCCCCATGCGGTAGAGATG
ATCAGCCGTCTCAGTGCCATGGGCATAGACGTCGAGACATGCCTCTGGGGCCAGCCTCTCCCCTCTTGCGACATCATTTCCGTCCTCGAGCTCGAGAAGCCTTTATTGCATGAAATGT
CCGAGGAGACCTTCAAGACCATCATTGCCTACTTCCAGAGCCACAAGGCAAGGGTGATCTGGGTGACCGAAGCATGCCAGATAGACTGCGCGAACCCCGAGCCTGCAATGATGCTCGG
CCTCGCCAGGACGGTCCGGAACGAATACTCACTACAGATGTACACCGTCGAGCTCGACAAAGGGACGACCATGAGCAGGGCCACCGAGGCCATCATTGACATCTGGGGCCGGGTCAGC
ACTCCGGACCTAGATCCGGAGTCTATGGATCACGACTACGAATATGCCTTGGTCGACGGCAGGATCCTGATTCCGCGATTCCACTGGCAGACGATGTCGGGTGCATTTTCAGGGGCCG
CGCGCAAGGAGACAGATGATGTCGAGGTCGCTCTCAAGCACCTCAACATGTCGACTCCAGGTCTTCTACGCACAATGAAGTGGATTGACGGCAGCGTTCCGACCGCTCCCGCCAAGGG
CGAGGTGTTAGTCGAAGTGAAGGCCGTTGGTCTCAACTTCAGGGATGTTATTCTGGCTCTGGGCGTAGTCGAAGGGAATCCCAGCGGAATGGGCCACGAGGGCAGCGGTGTCATCCGC
GCAGTCGGACCAGATGTCCAAGACCTCTCCGTTGGCGACAGGGTCATGTTCATCCACGACGGCTGCTTCACCACGCAGCTGACGTTGTCGAAGGACATCTGCGTCAGGCTCGACGACT
CGACGAGCTTTGTCCAGGGTGCCGCTCTGCCCGCCGTGCATGCAACCGCTCTGGCGGCACTGGTCGATGTTTCCAGGCTTCAAAGAGGACAGTCCGTGCTCATCCATGCCGCATGTGG
TGGCGTCGGTTTGGCCGCGATCCAGATTGCACAGAACATTGGTGCACAGATATACTGCACGGTAGGAAGCGAGCCGAAGCGGAGGTACCTCGAGGACACCTACAATATCCCGGCGGAC
CACATCTTCAACTCCCGCGACGTGACGTTCCTTCCAGAGGTCATGCGCGCGACCAACGGACGCGGCGTTGACGTCGTCCTCAACTCGCTCTCCGGCGACCTCCTGCACGCGTCGTGGA
AGTGCGTCGCCGAGTTTGGCCTGATGGTAGAGATTGGGAAACGCGACTTCCAGCGCCGGTCAAAGCTCGCCATGGAGGTGTTCGAGGCGAACCGGAGCTTCGTGGGCCTGGACATACG
CGGGCTTTCCATCTCTCGCCCGGAGAGGGCCGCCGATCTGATGCGTCGATGCGTCGACATGATCCGGTCGAGGGCCATCCAGGGTCCTGTCGCGTGCACAACCTTCCCGGCCGTCGAG
ATCCAGGATGCCTACAGGTACATGCAGAGCGCCAAGCATATCGGTAAGATTGTCATCGAGATGCCTGATGACCCGCGGGACCTTGGTGCAGGGGCAGCACCTCCAGAGGGCGAGTCGA
CCGAGCTGGAAGTCTCTCCCAGGCCCAAGCCCAGCTTCCGCCCCGATCGTAGCTATCTCCTCGTGGGTGGTTTGGGAGGGCTGGGACGTGCTGTGGCAACCTGGATGGTGGAACACGG
AGCGCGGGTCTTGGTCTTCCTCTCCAGATCGGCCCGGGAGAATGATGACAACCGAGACTTCCTCAACGACCTCCGCTCCGAAGGTTGCACTGTTCTGTTGGTTCCCGGAAGCGTCAGC
AACCTCGAGGATGTGCAAAAGGGGGTGGCAGTTGCTACGGCTGACCAGCCACTCGGCGGCCTCATCAACCTGTCCATGGTCCTCAGGGATGTCACGCTCAACAAGATGACATACTCGG
ACTGGACCACGGCCGTGGAGCCAAAGGTCAGGGGAACGTGGAATCTGCATCACGCCACTGCAAACTGTACTTCCTTGGAGTTCTTCATACTGTTCTCTTCACAAAACGCTCAGATCGG
CCAATGGGGCCAGGCCAACTACGCGGCTGCCAACACTTTCCTCGACGCCTTTGCCCAGTACCGGCACGGGCACAGCCTCGTCGCTTCGGTCATCGATGTCGGTCTCATGGGCGACGTG
GGCTTCGCGGCGGAGAACCGCGCGATCCTGAAGAAGCTGGGTAGGATCGGCATGTATATCCTGCAAGAGACAGACCTGCTCGATGCCATCAGCCTGGCTCTTCTGAAGTCACAGCCCA
TGCACGAGACGGAAGACAAGACGAGCCGCTACGTCACGCCGGGCTACGTGGGTATCGGCCTCAACACCACGACGCCCATGTCTGCCGCCTCGACCCGCGTCCCGTGGAAGCGAGACCC
GCGCATGAGCATCTACCACAACATGGATAACTCCAGCGGAGATGTCAGAGGCGAGGGCTCCTCCAAGGGCAGCTCGCTCAAGGTCGTCCTGGCCGCCGAGCCATCTGAGGAGAAGAAG
ACCGAGATCATCGCCAAGGCCCTCGCCGGCACTCTCGGGAACTTCCTCATCAAAGACGGGAGCAGCTTCCCACTCGACAAGCCCCTCAAGATGCTCGGCATGGATTCTTTGATCGCCA
TGGAGGTGCGGAACTGGATCCGACAGAACATCGGCGCCGAGACAAGCACGTTCACCGTGCTACAGAGCTCTTCCTTCATGCATCTGGCCGGCGAGATCAGGGCGGCGATGAATGCGGC
GAGCGAGGAGTGA
```

### 3.2. Amino Acid Sequences

**>SimG_hrPKS (residue numbering used in main text)**

```
        10         20         30         40         50         60
MAPQQISPPN GTASPEAGTP RYTYRPASPA PESPWADSAE GAELAKPMAI IGMAMRLPGS
        70         80         90        100        110        120
VRSGDDLWDL LSTRKSGLCD IPKNRFNADG FYDPARGPGT IPVKKGYFLH DVQIEEFDTN
       130        140        150        160        170        180
VFPIPKMELE RLDPAQRQLL QVAYECIENA GGTSWRGSRT GCYIGEFGED FADSSARESQ
       190        200        210        220        230        240
QRGNLRYTGL ADFAIANRIS YELDLQGPSM VVKTACSSSL VCLDLACKAI QSGECESALV
       250        260        270        280        290        300
GGVSMLFSPA TYISVTDLGV ISPNGQCRSF DAGADGYARG EAVNMVMIKR LDHALRDKDP
       310        320        330        340        350        360
IRAVIRGTGV NTDGRTNGML TPSSAVQADL IRHTYEIAGI DDLSQTAVVE CHGTGTPVGD
       370        380        390        400        410        420
PIETEAVGRC FGDDGVTITS VKPNLGHAEA AAGLSSLIKC VLSLEHRQVL PNINFETPNP
       430        440        450        460        470        480
DIPFEKYKLR VPTEVESWPQ GRAERISINA FGIGGVNAHA IIESPAQFGI KKPANGIAGR
       490        500        510        520        530        540
GVHTNGDSVP NGSQKQHEHD HRQDQSAYVK GDYMNGTAVN GANGNEKHAD RGPNLLVFSA
       550        560        570        580        590        600
YSSESLDRQI SAYRDFAATH QGASLKDLAY TLSSRRDHRP YRAYAIADDA SGVQDASATV
       610        620        630        640        650        660
NAVVDGAPPP VGWIFTGQGA QWPEMGARLI DTSAAFRNRI RKLDKYLQTL KLEPPVSIEA
       670        680        690        700        710        720
ELRKTKEDSR VHRPEMGHLV CVAMQVALVD VLRSWNIVPD FVLGHSSGET AAAYACGAIT
       730        740        750        760        770        780
AEAAVYAATR RGIGNASSQR KGSMAAVGLG RDEIQPFLLE GVNIACENSQ SNVTLSGDTE
       790        800        810        820        830        840
QVESIVATLK AERPGVFARL LRVEKAYHSH HMCEYGPVYE EQLKPVVRSA DPEIPFYSSV
       850        860        870        880        890        900
TGDRLTGEGQ LGASYWRANM ENTVLFNPAL RSALRDRPGK LVLIELGPHP ALEGPVGQIL
       910        920        930        940        950        960
RDLGRTDDIY LGTCIRGSEC DRSLLHLGGK LYQQSIPMDL AAICPPGAVL TNLPRYAWSQ
       970        980        990       1000       1010       1020
DTTHWEESRV SRGWRLREHP PHELLGSRVL ETEGEPCWRK VMALEDALWL DGHEVNGQVL
      1030       1040       1050       1060       1070       1080
FPAAGYISMV GEALRQLTGD ATFTVRNVRI MSGLVLSSDK PVELVTLLRS TTMGTSDDSE
      1090       1100       1110       1120       1130       1140
WYEFTITSFD GTAWVRNCHG EAMASSDKSF HLDSVSPAAG SFPRKLDRRD SYDILRRVGF
      1150       1160       1170       1180       1190       1200
NYTGEFEGMA DISVSPSSLQ AHGVTSPARQ VTDRPSRCAT YSVHPAVIDQ CFQLFTVALC
      1210       1220       1230       1240       1250       1260
RGLRRSNRRL AVPTFIEELV VSPSASPLSV TAKINRLDEV GSWTGDFVAL AAEGPVVRLK
      1270       1280       1290       1300       1310       1320
GMKSVVLATP AQSEPPLSTE LEWKPHSDFV GLGVGLHPRV PRKREWALLE ELVLLCNLDH
      1330       1340       1350       1360       1370       1380
LGQIKTDDST APYLIKLLDW MRLVTDRYRS GKNLFVSAGA GLEHLDHDER VARIDFLMAQ
      1390       1400       1410       1420       1430       1440
LADTQDVVFA RAIHRLFIEA PSIFAGEGHP LHILMPDNVL SDFYDATSFD MADAVRLVAN
      1450       1460       1470       1480       1490       1500
TNPHMRVLEV GAGTGSMTAR LLRALTSSHG ERLYSQYGYT DVSAGFMAVG KQRFAAVENI
      1510       1520       1530       1540       1550       1560
DYDVLDISKD PKEQGFRPGS YDLVIASNVL HATPTLDETL RNVYSLLKPS GRLFLEELTP
      1570       1580       1590       1600       1610       1620
DAMFVNYVMG FFYGWWLGAE DNRVEQPWIA PECWAEKLVA AGFQKPEAIV LDSDKPYQIS
      1630       1640       1650       1660       1670       1680
AGIIASRGVR STLPSKVAVL CHTADEPHAV EMISRLSAMG IDVETCLWGQ PLPSCDIISV
```

```
       1690       1700       1710       1720       1730       1740
LELEKPLLHE MSEETFKTII AYFQSHKARV IWVTEACQID CANPEPAMML GLARTVRNEY
       1750       1760       1770       1780       1790       1800
SLQMYTVELD KGTTMSRATE AIIDIWGRVS TPDLDPESMD HDYEYALVDG RILIPRFHWQ
       1810       1820       1830       1840       1850       1860
TMSGAFSGAA RKETDDVEVA LKHLNMSTPG LLRTMKWIDG SVPTAPAKGE VLVEVKAVGL
       1870       1880       1890       1900       1910       1920
NFRDVILALG VVEGNPSGMG HEGSGVIRAV GPDVQDLSVG DRVMFIHDGC FTTQLTLSKD
       1930       1940       1950       1960       1970       1980
ICVRLDDSTS FVQGAALPAV HATALAALVD VSRLQRGQSV LIHAACGGVG LAAIQIAQNI
       1990       2000       2010       2020       2030       2040
GAQIYCTVGS EPKRRYLEDT YNIPADHIFN SRDVTFLPEV MRATNGRGVD VVLNSLSGDL
       2050       2060       2070       2080       2090       2100
LHASWKCVAE FGLMVEIGKR DFQRRSKLAM EVFEANRSFV GLDIRGLSIS RPERAADLMR
       2110       2120       2130       2140       2150       2160
RCVDMIRSRA IQGPVACTTF PAVEIQDAYR YMQSAKHIGK IVIEMPDDPR DLGAGAAPPE
       2170       2180       2190       2200       2210       2220
GESTELEVSP RPKPSFRPDR SYLLVGGLGG LGRAVATWMV EHGARVLVFL SRSARENDDN
       2230       2240       2250       2260       2270       2280
RDFLNDLRSE GCTVLLVPGS VSNLEDVQKG VAVATADQPL GGLINLSMVL RDVTLNKMTY
       2290       2300       2310       2320       2330       2340
SDWTTAVEPK VRGTWNLHHA TANCTSLEFF ILFSSQNAQI GQWGQANYAA ANTFLDAFAQ
       2350       2360       2370       2380       2390       2400
YRHGHSLVAS VIDVGLMGDV GFAAENRAIL KKLGRIGMYI LQETDLLDAI SLALLKSQPM
       2410       2420       2430       2440       2450       2460
HETEDKTSRY VTPGYVGIGL NTTTPMSAAS TRVPWKRDPR MSIYHNMDNS SGDVRGEGSS
       2470       2480       2490       2500       2510       2520
KGSSLKVVLA AEPSEEKKTE IIAKALAGTL GNFLIKDGSS FPLDKPLKML GMDSLIAMEV
       2530       2540       2550       2560
RNWIRQNIGA ETSTFTVLQS SSFMHLAGEI RAAMNAASEE
```

Catalytic residues highlighted in Red and Ppant attachment site in Cyan.

**pHis$_8$-SimG AT**

```
MKHHHHHHHH SSGLVPRGSHM-

GNEKHADRGP NLLVFSAYSS ESLDRQISAY RDFAATHQGA SLKDLAYTLS SRRDHRPYRA
YAIADDASGV QDASATVNAV VDGAPPPVGW IFTGQGAQWP EMGARLIDTS AAFRNRIRKL
DKYLQTLKLE PPVSIEAELR KTKEDSRVHR PEMGHLVCVA MQVALVDVLR SWNIVPDFVL
GHSSGETAAA YACGAITAEA AVYAATRRGI GNASSQRKGS MAAVGLGRDE IQPFLLEGVN
IACENSQSNV TLSGDTEQVE SIVATLKAER PGVFARLLRV EKAYHSHHMC EYGPVYEEQL
KPVVRSADPE IPFYSSVTGD RLTGEGQLGA SYWRANMENT VLFNPALRSA LRDRPGKLVL
IELGPHPALE GPVGQILRDL GRTDDIYLGT CIRGSECDRS LLHLGGKLYQ QSIPMDLAAI
CPPGAVLTNL PRYAWSQDTT HWEESR-
```

MW (including His-tag) = 51,161.72 Da

Catalytic Ser and His residues highlighted in Red.

**pHis$_8$-SimG_ACP**

```
MKHHHHHHHH SSGLVPRGSHM-

GEGSSKGSSL KVVLAAEPSE EKKTEIIAKA LAGTLGNFLI KDGSSFPLDK PLKMLGMDSL
IAMEVRNWIR QNIGAETSTF TVLQSSSFMH LAGEIRAAMN AASEE-
```

MW (including His-tag) = 13623.56 Da

Ppant attachment site highlighted Cyan.

**pHis8-SimG KS-AT**

MKHHHHHHHH SSGLVPRGSHM-

APQQISPPNG TASPEAGTPR YTYRPASPAP ESPWADSAEG AELAKPMAII GMAMRLPGSV
RSGDDLWDLL STRKSGLCDI PKNRFNADGF YDPARGPGTI PVKKGYFLHD VQIEEFDTNV
FPIPKMELER LDPAQRQLLQ VAYECIENAG GTSWRGSRTG CYIGEFGEDF ADSSARESQQ
RGNLRYTGLA DFAIANRISY ELDLQGPSMV VKTACSSSLV CLDLACKAIQ SGECESALVG
GVSMLFSPAT YISVTDLGVI SPNGQCRSFD AGADGYARGE AVNMVMIKRL DHALRDKDPI
RAVIRGTGVN TDGRTNGMLT PSSAVQADLI RHTYEIAGID DLSQTAVVEC HGTGTPVGDP
IETEAVGRCF GDDGVTITSV KPNLGHAEAA AGLSSLIKCV LSLEHRQVLP NINFETPNPD
IPFEKYKLRV PTEVESWPQG RAERISINAF GIGGVNAHAI IESPAQFGIK KP**ANGIAGRG**
**VHTNGDSVPN GSQKQHEHDH RQDQSAYVKG DYMN**GTAVNG ANGNEKHADR GPNLLVFSAY
SSESLDRQIS AYRDFAATHQ GASLKDLAYT LSSRRDHRPY RAYAIADDAS GVQDASATVN
AVVDGAPPPV GWIFTGQGAQ WPEMGARLID TSAAFRNRIR KLDKYLQTLK LEPPVSIEAE
LRKTKEDSRV HRPEMGHLVC VAMQVALVDV LRSWNIVPDF VLGHSSGETA AAYACGAITA
EAAVYAATRR GIGNASSQRK GSMAAVGLGR DEIQPFLLEG VNIACENSQS NVTLSGDTEQ
VESIVATLKA ERPGVFARLL RVEKAYHSHH MCEYGPVYEE QLKPVVRSAD PEIPFYSSVT
GDRLTGEGQL GASYWRANME NTVLFNPALR SALRDRPGKL VLIELGPHPA LEGPVGQILR
DLGRTDDIYL GTCIRGSECD RSLLHLGGKL YQQSIPMDLA AICPPGAVLT NLPRYAWSQD
TTHWEESR

MW (including His-tag) = 106,963.24 Da (full length); 102,404.44 Da (Δ473-514).

Catalytic Cys and His residues highlighted in Red.

Removed linker residues highlighted in Grey – gives rise to SimG KS-AT(Δ474-515).

## 3.2. Supplementary Tables

**Table S1.** Primers for cloning of pHis$_8$-SimG.

| Construct | Forward Primer (5'-3') | Reverse Primer (5'-3') |
|---|---|---|
| pXW55-SimG | CCATCACCATCACCATCACCATCACACTAGTG CTCCTCAACAAATATCACCCCCCAATGG | CAAGCTCAGGACACACTTGATCAGGCTGG ACAG |
| | CTGTCACAAACCGCCGTCGTGGAGTGTC | GGTTGAAGAGAACCGTGTTCTCCATGTTCG |
| | GAGATCCCATTCTACTCCTCCGTCACCGGTG | CTGTCCAGAACGATGGCTTCGGGCTTCTG |
| | CATATCGAAGGATCCCAAGGAGCAGGGATTCA G | GATAATGAAAACTATAAATCGTGAAGGCAT TCACTCCTCGCTCGCCGCATTCATCGCCG C |

**Table S2.** NMR Data for 3R-hydroxyl-4R-methyl-6E-octenoic acid (Hma, **3**)

| | | |
|---|---|---|
| | 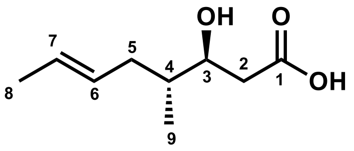  Data in agreement with reported literature.[2] | |
| Pos. | $\delta_C$ | $\delta_H$, multi, (*J* in Hz) |
| 1 | - | |
| 2 | 38.2, $CH_2$ | Ha: 2.63 – 2.49, m, 1H<br>Hb: 2.49 – 2.37, m, 1H |
| 3 | 71.8, CH | 3.87, br, 1H |
| 4 | 38.7, CH | 1.66, overlap, 1H |
| 5 | 35.9, $CH_2$, | Ha: 2.21 – 2.13, m, 1H<br>Hb: 1.89, dt, *J* = 14.3, 7.6, 7.6 Hz, 1H |
| 6 | 129.1, CH | 5.52 – 5.35, overlap, 1H |
| 7 | 127.2, CH | 5.52 – 5.35, overlap, 1H |
| 8 | 18.1, $CH_3$ | 1.66, d, *J* = 5.6 Hz |
| 9 | 15.2, $CH_3$ | 0.88, d, *J* = 6.6 Hz |

**Table S3.** Primers, annealing temperatures and restriction sites for cloning and mutagenesis of SimG KS-AT, AT and ACP constructs.

| Construct | Forward Primer (5'-3') | Reverse Primer (5'-3') | Temp. (°C) |
|---|---|---|---|
| **pHis$_8$-SimG KS-AT** | ATA**CATATG**GCTCCTCAACAAATATCA (NdeI) | ATA**AAGCTT**TCACCTGGATTCTT (HindIII) | 52 |
| **pHis$_8$-SimG AT** | ATA**CATATG**GGCAACGAAAAGCAT (NdeI) | ATA**AAGCTT**TCACCTGGATTCTT (HindIII) | 69 |
| **pHis$_8$-SimG ACP** | ATA**CATATG**GGCGAGGGCTCCTCCAA (NdeI) | ATA**AAGCTT**TCACTCCTCGCTCGC CGCATT (HindIII) | 69 |
| **Mutation** | **Forward Primer (5'-3')** | **Reverse Primer (5'-3')** | **Temp. (°C)** |
| **pHis$_8$-SimG KS-AT (Δ473-514)** | GGCACTGCCGTCAACGGG | AGGCTTCTTGATTCCAAACTGCG | 68 |
| **pHis$_8$-SimG KS-AT$^0$ (Δ473-514), (S705A)** | **AT$^0$**<br>ATAGGCAACGAAAAGCATGC<br>**pET28a(+)_KS**<br>ATAACGACGCACTGGGAAGAATCCA | **AT$^0$**<br>ATATCACCTGGATTCTTCCCA<br>**pET28a(+)_KS**<br>ATATCCGCGGTCAGCATGCTTTT | 70 |
| **pHis$_8$-SimG KS$^0$-AT$^0$ (Δ473-514), (C215A, S705A)** | CAAGACGGCTGCATCGTCTTCCCTCGTG TG | ACCACCATACTCGGGCCT | 65 |

## REFERENCES


1 J. Abramson, J. Adler, J. Dunger, R. Evans, T. Green, A. Pritzel, O. Ronneberger, L. Willmore, A. J. Ballard, J. Bambrick, S. W. Bodenstein, D. A. Evans, C.-C. Hung, M. O'Neill, D. Reiman, K. Tunyasuvunakool, Z. Wu, A. Žemgulytė, E. Arvaniti, C. Beattie, O. Bertolli, A. Bridgland, A. Cherepanov, M. Congreve, A. I. Cowen-Rivers, A. Cowie, M. Figurnov, F. B. Fuchs, H. Gladman, R. Jain, Y. A. Khan, C. M. R. Low, K. Perlin, A. Potapenko, P. Savy, S. Singh, A. Stecula, A. Thillaisundaram, C. Tong, S. Yakneen, E. D. Zhong, M. Zielinski, A. Žídek, V. Bapst, P. Kohli, M. Jaderberg, D. Hassabis and J. M. Jumper, *Nature*, 2024, **630**, 493–500.

2 A. Rittner, K. S. Paithankar, A. Himmler and M. Grininger, *Protein Sci*, 2020, **29**, 589–605.

3 C. J. B. Harvey, M. Tang, U. Schlecht, J. Horecka, C. R. Fischer, H. C. Lin, J. Li, B. Naughton, J. Cherry, M. Miranda, Y. F. Li, A. M. Chu, J. R. Hennessy, G. A. Vandova, D. Inglis, R. S. Aiyar, L. M. Steinmetz, R. W. Davis, M. H. Medema, E. Sattely, C. Khosla, R. P. S. Onge, Y. Tang and M. E. Hillenmeyer, *Sci. Adv.*, DOI:10.1126/sciadv.aar5459.
